# Effects of non-pharmacological interventions on sleep in premature infants: meta-analysis

**DOI:** 10.1590/1806-9282.20241187

**Published:** 2024-12-02

**Authors:** Fatma Şule Bilgiç, Aysu Yıldız Karaahmet, Berker Okay

**Affiliations:** 1 Haliç University, Faculty of Health Sciences, Department of Midwifery – İstanbul, Turkey.; 2 Haseki Training and Research Hospital, Child Health and Diseases Unit – İstanbul, Turkey.

## INTRODUCTION

Premature birth is a consequence of various unforeseen circumstances. Babies born before the thirty-seventh week of gestation are described as "premature." Preterm birth is among the most important causes of mortality and morbidity in infants^1,2^. Premature infants (PI) experience different stimuli such as light exposure, intense noise, stressful, and painful interventions during their stay in the neonatal intensive care unit^3,4^.

Considering that diaper changes occur only eight times a day with painful procedures for diagnosis and treatment, these stimuli can affect the cognitive and behavioral development of the premature baby. This is reflected in physiological parameters such as heart rate (HR), oxygen saturation (SpO_2_), salivary cortisol, and behavioral parameters such as changes in sleep–wakefulness, facial expressions, and crying^5,6^. These problems adversely affect the neurodevelopment and stability of the premature baby. Neurodevelopmental and neurological problems are the most important morbidities that occur after premature birth^5^.

Evaluation of the behavioral status of premature babies is important in terms of determining and supporting behavioral regulation skills. Approaches and care models that support the baby's neurological development are proposed in the prevention of an early identifiable behavioral or developmental disorder^6^. Identifying and applying appropriate approaches specific to behavioral status can positively affect neurodevelopment^5,7^.

## METHODS

In this study, it was aimed to systematically review the sleep and physiological parameter results of interventions in preterm infants and to conduct a meta-analysis of the available evidence. PRISMA (Preferred Reporting Items for Systematic Reviews and Meta-Analyses statement) was followed in the preparation of the systematic review and meta-analysis. The protocol for this systematic review and meta-analysis is recorded in the PROSPERO database.

### Eligibility criteria

The following criteria (PICOS) were taken into account in the selection of the studies to be included in the study:

- Participant (P): Preterm and very low birth weight infants. Infants included in the study had the following inclusion criteria: (1) Infants born before 37 weeks gestational age; (2) without a history of cerebral hemorrhage, neurological and central nervous system disease, and convulsions; and (3) infants not on sedative medication.
- Intervention (I): Pharmacological and nonpharmacological methods. Nonpharmacological methods: (1) Hammock; (2) Nest, pharmacological methods: (1) High-protein diet in parenteral and enteral period.
- Comparison (C): (1) Traditional position and standard protein delivery; (2) Crossover study.
- Outcomes (O): (1) Sleep duration; (2) Sleep–wake states.
- Study design (S): Randomized controlled trials were included. Non-preterm articles that did not evaluate sleep and physiological parameters in their results were excluded from traditional and systematic reviews.

### Search strategy

The literature review for this systematic review was conducted between May and June 2024 using five electronic databases (PubMed, CINAHL, Scopus, WOS, and ULAKBİM). The studies in which the efficacy of interventions in preterm babies were examined were screened by selecting keywords. The keywords were "preterm" OR "prematurity" OR "baby" OR "newborn" OR "infant" AND "therapy" OR "nonpharmacological" OR "pharmacological" OR "alternative therapy" AND "sleep" AND "physiologic parameters." The search strategy was changed according to the characteristics of each database. In addition, reviews on articles included in systematic reference lists and other previous systematic reviews were checked to reach further studies.

### Selection of studies and data extraction

After removing duplicate articles from different databases, two researchers (AYK and FŞB) independently conducted literature review, article selection, data extraction, and quality evaluation of the included articles to control the risk of bias during the study. Full texts that met the inclusion criteria but could not be identified in the title/abstract scan were examined. In studies where consensus could not be reached, the researchers considered working as partners. A data extraction tool developed by the researchers was used to obtain the research data. Two researchers (AYK and FŞB) analyzed the study according to place and year, publication year, research design, sample size, and the effect of interventions on sleep (Table 1).

**Table 1:** Studies characteries (n=5).

Author/Country	Study design	Population	Groups	Gestational week (mean±SD)	Birth weight (mean±SD)	Sleep (mean±SD)	Heart rate (mean±SD)
Hammond et al.^12^; New York	Double-blind RCT	71 preterm infants (IG: 37; CG: 34)	IG: High protein group CG: Conventional group	IG: 26.8±2.1 CG: 26.8±1.8	IG: 938.9±219.65 CG: 937.9 ±230.4	IG: 70.7±11.8 CG: 77.2±10.5	NA
Costa et al.^11^; Brazil	Crossover RCT	20 preterm infants (IG: 6; CG: 14)	IG: Hammock-positioning CG: Neck positioning	IG: 31.4±2.17	1.39±1.23	IG: 30.00±14.29 CG: 27.00±19.05	IG: 151.10±19.526 CG: 143.15±37.406
Ribas et al.^13^; Brazil	RCT	26 preterm infants (IG: 13, CG: 13)	IG: Hammock-positioning CG: Traditional-positioning	IG: 33.1±1.0 CG: 32.31±1.70	IG: 1.31±0.48 CG: 1.62±0.51	IG: 1.23±0.44 CG: 2.08±0.64	IG: 142.77±5.18 CG: 151.69±5.44
Kobus et al.^9^; Germany	RCT	40 preterm infants (IG: 20, CG: 20)	IG: Therapy group CG: Control group	IG: 28.6±2.4 CG: 28.9±2.7	IG: 1059±389 CG: 1201±404	155.5 (152.2–158.8)	14.2 (95% CI 17.5–10.9)
Düken et al.^8^; Turkey	RCT	120 preterm infants (IG: 40; IG2: 40;CG: 40)	IG: Massage group IG2: White noise group CG: Control group	IG: 26.7±5.8 IG2: 26.2±6.5 CG: 26.6±6.6	IG: 1942.6±240.0 IG2: 1951.6±187.8 CG: 1958.8±262.1	IG: 86.8±9.2 IG2: 86.5±8.9 CG: 88.4±10.5	IG: 66.3±56.5 IG2: 58. 4±45.9 CG: 65.2±57.9

IG: ıntervention group; CG: control group; SD: standard deviation; RCT: randomized controlled trial.

### Evaluation of the methodological quality of the studies

The quality of the articles in randomized controlled trials and Version 2 of the Cochrane Risk-of-Bias tool (RoB-2) were used for randomized trials.

### The data analysis

Meta-analysis was performed using Review Manager 5.4 (The Nordic Cochrane Center, Copenhagen, Denmark) for data analysis. The heterogeneity between the studies was evaluated using Cochran's Q test and Higgins’ I², and it was accepted that I² greater than 50% showed significant heterogeneity. Accordingly, random effect results were taken into account when I² was greater than 50%, and fixed effect results were taken into account if it was less than the value. Odds ratio for categorical variables, mean difference (MD), and standardized mean difference (SMD) for continuous variables were calculated. MD or SMD, along with the corresponding 95% confidence interval (CI), is appropriately pooled for continuous variables based on whether the results are measured on the same scales.

## RESULTS

### Literature review

**Figure 1:**
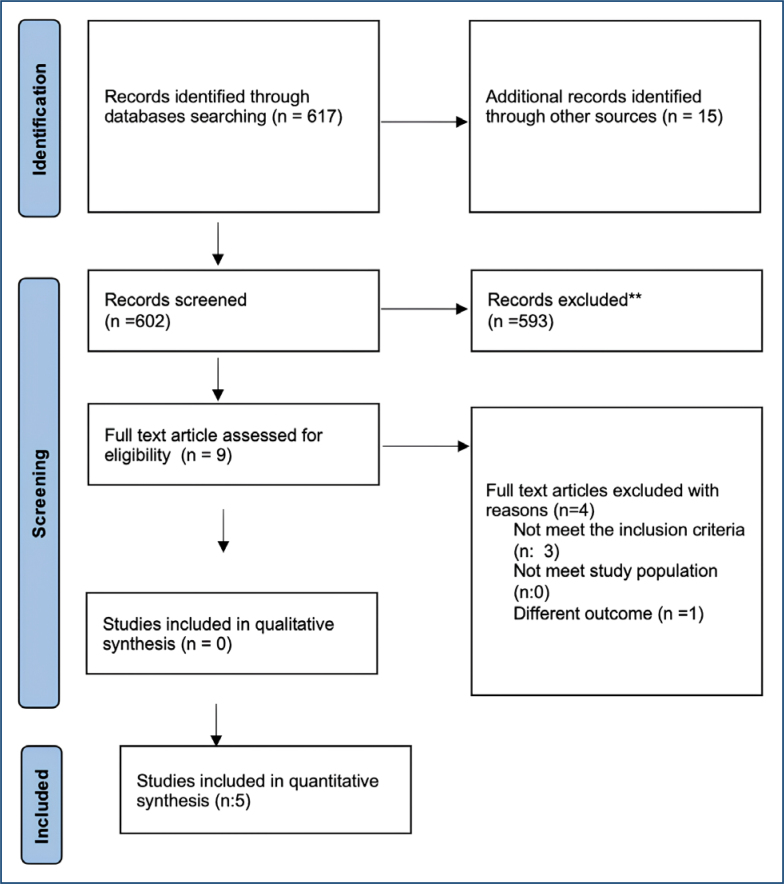
PRISMA flow diagram.

The PRISMA flowchart for literature review and selection is summarized in Figure 1. A total of 15 studies were reached through electronic database research and manual search. A total of 2,617 articles, the full text of which can be accessed, were examined. Titles and abstracts were read to identify the relevant articles, and 15 articles were removed because they did not meet the criteria for review articles, protocols, duplications, different populations, and inclusion. The remaining six full texts were evaluated for eligibility. Five randomized controlled trial (RCT) articles were included in the quantitative synthesis because they met the desired criteria (see Figure 1). The five RCTs included outline the study (Table 1). RCTs were ranked chronologically from the nearest date to the farthest.

### Study characteristics

This systematic review and meta-analysis included five studies in which a total of 238 preterm infants were included to assess the impact of interventions on sleep duration, sleep–wake states, and outcomes related to physiological parameters in preterm infants^8–10^. All the studies included in the meta-analysis were RCT studies. The characteristics of the studies are summarized in Table 1. In the interventions for preterm infants in the articles included in the study, Costa et al.^11^ applied nest and hammock, Hammond et al.^12^ applied high-protein therapy and diet, and Ribas et al.^13^ applied hammock. The intervention time of the studies in the review varies. Hammond et al.^8^ evaluated very low birth weight preterm infants in two phases: total parenteral nutrition and enteral feeding period, and the time between phases differed according to the condition of the babies. Costa et al.^11^ evaluated 5 min before the intervention, 1 min after the baby's diaper was changed and the nest/hammock was placed, followed by 5 and 10. Evaluation was made in minutes. In Ribas et al.^13^ infants were evaluated 10 min before and immediately after the 2-h intervention for 5 consecutive days. The pattern of Costa et al.^11^ is crossover, in which both groups were intervened. In the study of Hammond et al.^8^ the control group received conventional protein supplementation, while in the study of Ribas et al.,^13^ the control group remained in the traditional position. The interventions made to preterm babies in the intervention group, Ribas et al.,^13^ applied the hammock side lying position for 2 h every day for 5 consecutive days to preterm infants with gestational age at 30–37 weeks Hammond et al.^8^ Preterm infants weighing between 500 and 1,250 g were given 18% of their gross energy intake as protein in phase 1 of the parenteral period (protein:energy ratio: 4.5 g:100 kcal). When Phase 2 started, protein intake in this group was fixed at 4 g/kg/day (0.004 kcal/kg/day). Costa et al.^11^ gave one group a hammock position and a nest position in the other group in their study. While sleep–wake status and sleep duration were evaluated in the studies, physiological parameters were evaluated in Costa et al.^11^ and Ribas et al.^13^ studies. In the study of Düken et al^8^, massage and white light were evaluated compared to the control group. In the study of Kobus et al^9^, sleep status was evaluated by applying music therapy to PI.

### Outcomes

In the included research, the effect of interventions applied to preterm infants on physiological parameters and sleep status was examined.

### Sleep

**Figure 2:**
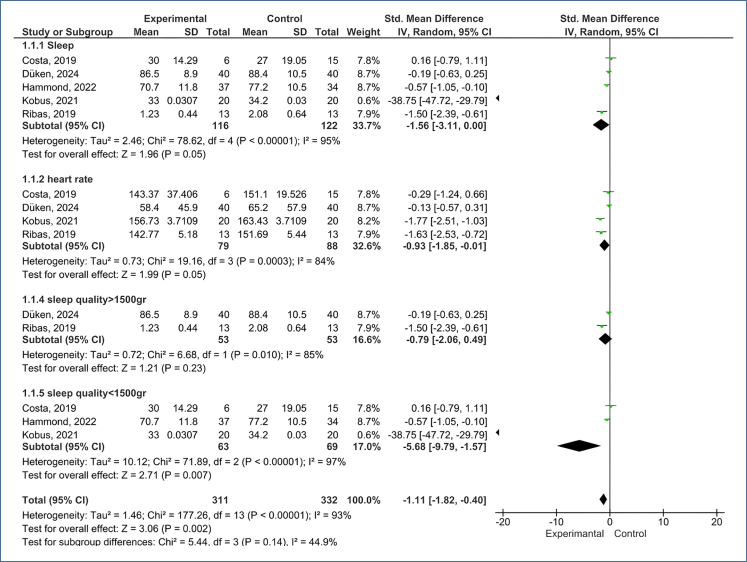
Mean analysis results including changes in nonpharmacological interventions according to sleep duration, quality, heart rate, and baby weight variables.

In all the studies reviewed, the authors presented data on the effect of non-pharmacological methods on sleep quality. The pooled standard mean results of the studies show that there is a difference in sleep quality between the groups (SMD: −1.56; 95%CI −3.11 to 0.00; Z=1.96; p=0.05) (Figure 2, 1.1.1). In the subgroup analysis made with the birth weights of the babies, sleep quality was more significant in babies weighing more than 1,500 g than in babies weighing less than 1,500 g (SMD: −5.68; 95%CI −9.79 to −1.57; Z=2.71; p=0.007) (Figure 2, 1.1.5).

### Heart rate

In four of the studies reviewed; the authors presented data on the effect of non-pharmacological methods on HR. The pooled standard mean results of the studies show that there is a difference in HR between the groups (SMD: −0.93; 95%CI −1.85 to −0.01; Z=1.99; p=0.05) (Figure 2, 1.1.2).

### Risk of bias assessment

The articles included in the study were assessed with the RoB-2 tool. While no high level of bias was observed in the studies, some concerns about bias were seen in one study, and a low risk of bias was observed in the other three studies.

## DISCUSSION

This systematic review and meta-analysis study was conducted to reveal the effect of non-pharmacological interventions applied to preterm infants in intensive care units on sleep and physiological parameter outcomes. The study results showed that non-pharmacological interventions added to the care of preterm infants had a significant effect on sleep quality and physiological parameters such as HR. Furthermore, non-pharmacological interventions were found to be more significant in infants weighing less than 1,500 g. This is a small result that shows that low-cost and high-impact behavioral interventions can be used to improve and maintain the health of infants.

The results of the study showed that the interventions made by adding to the routine care of preterm infants had increased sleep and wakefulness values between the groups compared to the control group, but the difference between them was not significant. A study showed that music therapy applied to preterm infants improved their sleep quality. Another study assessing the sleep–wakefulness of babies given white noise showed that babies’ sleep–wake was no different from routine care. The potential effects of sleep promotion can be influenced by a variety of factors, such as the baby's sleeping position, small sample size, and environmental stimuli. Sleep and wakefulness states affect the behavior, physiology, and ability to respond to stimuli of the premature baby^9,10^. Therefore, more studies with larger sample sizes focusing on these interventions aimed at improving sleep states are needed.

The results of the study showed that the interventions made by adding them to the routine care of preterm infants showed improvement in the physiologic parameter values between the groups compared to the control group, but the difference between them was not significant. When we look at the literature, there are studies that show that non-interventional interventions applied to preterm babies stabilize the vital signs of babies^14,15^. In one study, it was found that music therapy in preterm babies increased the oxygen saturation of newborns and decreased the pulse rate^16^. Non-interventional interventions to be applied to preterm babies are very important in terms of supporting the vital functions of babies.

The results of the study found that the difference between the interventions added to the routine care of preterm infants compared with the control group of physiological parameters was significant. In all the studies included in the study, HR was found to increase compared to the control group, while HR and respiratory rate decreased. In a meta-analysis involving 34 studies, it was reported that massage intervention applied to preterm infants positively affected the physiological parameters of preterm infants and had a significant difference compared to controls^15,16^. In another systematic review, kangaroo care did not show significant changes in the physiological parameters monitored^17,18^. These findings differ because the interventions applied to preterm infants are different from each other.

## CONCLUSION

In addition to the protective effect of non-interventional interventions or dietary changes against a wide range of negative neonatal outcomes, no evidence of harm has been shown. These safe, low-cost interventions have the potential to prevent many complications associated with premature birth, reduce intensive care unit admission rates, and may benefit preterm newborns.
